# Dose-dependent effect of daptomycin on the artificial prolongation of prothrombin time in coagulation abnormalities: in vitro verification

**DOI:** 10.1186/s40360-017-0180-3

**Published:** 2017-11-28

**Authors:** Hideki Hashimoto, Makoto Saito, Naoki Kanda, Takehito Yamamoto, Makiko Mieno, Shuji Hatakeyama

**Affiliations:** 1 Department of Infectious Diseases, The University of Tokyo Hospital, 7-3-1 Hongo, Bunkyo-ku, Tokyo, 113-8655 Japan; 2 Division of General Internal Medicine, Jichi Medical University Hospital, 3311-1 Yakushiji, Shimotsuke-shi, Tochigi, 329-0498 Japan; 3 Department of Pharmacy, The University of Tokyo Hospital (Current affiliation: The Education Center for Clinical Pharmacy, Graduate School of Pharmaceutical Sciences, The University of Tokyo), 7-3-1 Hongo, Bunkyo-ku, Tokyo, 113-8655 Japan; 4 Department of Medical Informatics, Center for Information, Jichi Medical University, 3311-1 Yakushiji, Shimotsuke-shi, Tochigi, 329-0498 Japan; 5 Division of Infectious Diseases, Jichi Medical University Hospital, 3311-1 Yakushiji, Shimotsuke-shi, Tochigi, 329-0498 Japan

**Keywords:** Daptomycin, Prothrombin time, Coagulation abnormality, Warfarin, Liver cirrhosis, False positive

## Abstract

**Background:**

Several studies have reported that daptomycin induced artificial prolongation of prothrombin time (PT) in some test reagents, particularly in warfarin users. However, it remains unknown whether the artificial prolongation can be affected by coagulation abnormalities other than the use of warfarin. Thus, we investigated the effect of daptomycin on PT with two types of coagulation abnormalities.

**Methods:**

Plasma samples were pooled by four groups: healthy volunteers (Plasma A), warfarin users with a PT-international normalized ratio (INR) of approximately 2.0 (Plasma B) or 3.0 (Plasma C), and patients with liver cirrhosis with a PT-INR of approximately 2.0 (Plasma D). Plasma A was composed of plasma from two healthy individuals (9 mL from each individual). Plasma B, C, and D were composed of plasma from 36 patients (0.5 mL from each patient). Daptomycin was added to each sample to create solutions with several concentrations (0–150 μg/mL). The PT-INR for each solution was measured with three PT reagents. Linear regression analyses were used to determine the association between daptomycin concentration and PT-INR. The relative change in PT-INR due to daptomycin concentrations was calculated.

**Results:**

Strong linear correlations were observed between daptomycin concentrations and PT-INR for all the plasma groups and reagents (R^2^ > 0.7, *P* < 0.01). At a daptomycin concentration of 150 μg/mL, the relative increase of PT-INR was ≥10% in the majority of the plasma groups with an elevated baseline PT-INR in all reagents tested.

**Conclusions:**

Daptomycin induced the artificial prolongation of PT-INR in a concentration-dependent manner, particularly in plasma samples with an elevated baseline PT-INR. PT should be evaluated at the trough levels of daptomycin.

## Background

Daptomycin is a cyclic lipopeptide antibiotic that is used to treat Gram-positive bacteria, such as methicillin-resistant *Staphylococcus aureus* (MRSA) [1]. It is inserted into the cytoplasmic membrane of Gram-positive bacteria and then disrupts the membrane, causing bacterial death. Daptomycin was approved by the United States Food and Drug Administration (FDA) in 2003 for the treatment of complicated skin and soft tissue infections and *S. aureus* bloodstream infections. Currently, it is also recommended for many serious infections caused by Gram-positive bacteria [2].

Some studies have reported the prolongation of prothrombin time (PT) in daptomycin users [3–5]. This prolongation is considered to be artificial because patients did not have actual bleeding tendencies, and it was observed when specific recombinant thromboplastin reagents were used to examine PT [3]. PT prolongation was concentration dependent, and therefore, the FDA suggests that PT should be evaluated at the trough levels of daptomycin [6].

Since this prolongation was not previously evaluated clinically, we did a prospective in vivo study to determine the effect of the daptomycin-induced PT prolongation in clinical settings using nine PT reagents that are available in Japan. Using several specific reagents, we confirmed that PT was significantly prolonged when daptomycin concentrations were high, particularly in warfarin users [7]. However, in vitro studies in a more controlled condition should be conducted to verify the results of our in vivo study. In addition, it remains unknown whether this prolongation is similarly observed in patients with other coagulation disorders, such as liver cirrhosis. Therefore, we conducted an in vitro study to determine whether daptomycin affects the PT-international normalized ratio (INR) in patients with coagulation abnormalities in addition to warfarin users.

## Methods

### Study population and setting

This study was based on pooled plasma samples from four groups: healthy volunteers (Plasma A), patients who took warfarin and whose PT-INR values were approximately 2.0 (Plasma B) or 3.0 (Plasma C), and patients who had liver cirrhosis and whose PT-INR values were approximately 2.0 (Plasma D). Patients who had liver cirrhosis and took warfarin were excluded in addition to patients with antiphospholipid syndrome. Plasma from two healthy individuals’ plasma was used for Plasma A. Plasma B, C, and D consisted of 36 patients’ plasma each. All patients were outpatients who visited the Jichi Medical University Hospital between June 2016 and August 2016.

### Sample preparation

Plasma samples were collected from 108 patients (0.5 mL each) and two healthy individuals (9 mL each) and stored at −80 °C. After all the samples were collected, frozen plasma samples were thawed and pooled by group. Daptomycin was added to each group of plasma samples to obtain 0, 5, 15, 50, 100, and 150 μg/mL solutions. Daptomycin was purchased from Merck Sharp & Dohme (Tokyo, Japan).

We examined the PT-INR of these solutions using three reagents. Reagent 1 (HemosIL RecombiPlasTin 2G: a recombinant human thromboplastin) was purchased from Werfen Japan (Tokyo, Japan). Reagent 2 (Neoplastin plus: a rabbit brain thromboplastin) and Reagent 3 (STA Neoplastin R: a recombinant human thromboplastin) were purchased from Roche Diagnostics K. K. (Tokyo, Japan). The international sensitivity index for each reagent was 0.97, 1.29, and 0.94, respectively.

### Data collection and statistical analyses

The PT-INR of all plasma samples were measured three times. The mean and standard deviation (SD) were calculated. Linear regression analyses for each plasma group and reagent were used to assess the associations between daptomycin concentrations and PT-INR. *P*-values less than 0.05 were considered statistically significant. Statistical analyses were performed using JMP Pro 12 (SAS Institute, Cary, North Carolina, USA). In addition, we calculated the relative change in PT-INR between high daptomycin concentrations (50, 100, and 150 μg/mL) versus the control (0 μg/mL) in each plasma group. We regarded 10% of the relative change in PT-INR as clinically significant, as described in previous studies [3, 7].

## Results

**Fig. 1:**
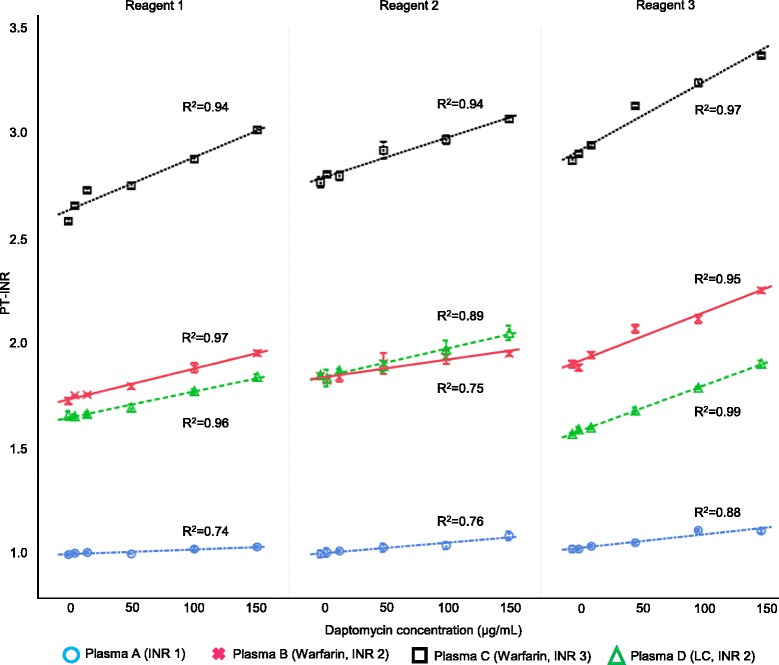
Linear regression analysis of daptomycin concentration and PT-INR for each reagent and plasma group. Data are presented as the mean and standard deviation. PT: prothrombin time, INR: international normalized ratio, LC: liver cirrhosis

We found strong and significant linear correlations between daptomycin concentrations and PT-INR for all the plasma groups and reagents (R^2^ > 0.7, *P* < 0.01 for all the groups and reagents) (Table 1 and Fig. 1). When the daptomycin concentration was 100 μg/mL, the PT-INR was prolonged by more than 10% in Plasma C using Reagent 1 and Plasma B, C, and D, respectively, using Reagent 3 (Table 2). At a daptomycin concentration of 150 μg/mL, almost all the relative changes in PT-INR in Plasma B, C, and D were more than 10% for all reagents. The maximum PT-INR prolongation was 22% when the daptomycin concentration was 150 μg/mL in Plasma D using Reagent 3 (Table 2).

**Table 1:** Results of the linear regression analysis of daptomycin concentrations and PT-INR

Reagent	Plasma group	R^2^	β	SE (β)	*P*-value
1: HemosIL RecombiPlasTin 2G	A (INR 1)	0.74	2.2 × 10^−4^	3.3 × 10^−5^	< 0.0001
	B (warfarin, INR 2)	0.97	1.5 × 10^−3^	6.0 × 10^−5^	< 0.0001
	C (warfarin, INR 3)	0.94	2.5 × 10^−3^	1.5 × 10^−4^	< 0.0001
	D (LC, INR 2)	0.96	1.3 × 10^−3^	6.5 × 10^−5^	< 0.0001
2: Neoplastin Plus	A (INR 1)	0.76	5.1 × 10^−4^	7.3 × 10^−5^	< 0.0001
	B (warfarin, INR 2)	0.75	8.6 × 10^−4^	1.2 × 10^−4^	< 0.0001
	C (warfarin, INR 3)	0.94	1.9 × 10^−3^	1.3 × 10^−4^	< 0.0001
	D (LC, INR 2)	0.89	1.4 × 10^−3^	1.2 × 10^−4^	< 0.0001
3: STA Neoplastin R	A (INR 1)	0.88	6.6 × 10^−4^	6.0 × 10^−5^	< 0.0001
	B (warfarin, INR 2)	0.95	2.4 × 10^−3^	1.3 × 10^−4^	< 0.0001
	C (warfarin, INR 3)	0.97	3.3 × 10^−3^	1.6 × 10^−4^	< 0.0001
	D (LC, INR 2)	0.99	2.2 × 10^−3^	4.7 × 10^−5^	< 0.0001

*INR* international normalized ratio, *SE* standard error, *LC* liver cirrhosis

**Table 2:** Baseline PT-INR values and ratios of PT-INR to the baseline PT- INR at designated daptomycin concentrations

Reagent	Plasma group	Baseline PT-INR (mean ± SD)	PT-INR_50_/PT-INR_0_^a^	PT-INR_100_/PT-INR_0_^a^	PT-INR_150_/PT-INR_0_^a^
1: HemosIL RecombiPlasTin 2G	A (INR 1)	0.96 ± 0.00	1.01	1.03	1.04
	B (warfarin, INR 2)	1.70 ± 0.01	1.04	1.09	1.14
	C (warfarin, INR 3)	2.57 ± 0.01	1.07	1.11	1.17
	D (LC, INR 2)	1.63 ± 0.02	1.02	1.07	1.11
2: Neoplastin Plus	A (INR 1)	0.97 ± 0.02	1.03	1.05	1.09
	B (warfarin, INR 2)	1.82 ± 0.01	1.03	1.05	1.06
	C (warfarin, INR 3)	2.75 ± 0.02	1.06	1.07	1.11
	D (LC, INR 2)	1.83 ± 0.00	1.02	1.07	1.11
3: STA Neoplastin R	A (INR 1)	0.99 ± 0.02	1.03	1.09	1.09
	B (warfarin, INR 2)	1.88 ± 0.02	1.09	1.12	1.19
	C (warfarin, INR 3)	2.86 ± 0.02	1.09	1.13	1.18
	D (LC, INR 2)	1.54 ± 0.01	1.07	1.14	1.22

*PT* prothrombin time, *INR* international normalized ratio, *SD* standard deviation, *LC* liver cirrhosis

^a^ PT-INR_0_ represents the baseline PT-INR. PT-INR_50_, PT-INR_100_, and PT-INR_150_ represent the PT-INR at daptomycin concentrations of 50, 100, and 150 μg/mL, respectively. The relative change in PT-INR was calculated using the mean values of PT-INR measured in triplicate at each daptomycin concentration

For Reagents 1 and 3, the relative increase in PT-INR appeared to be greater in the plasma groups with a high baseline PT-INR (Plasma B, C, and D) than in Plasma A with a normal baseline PT-INR (Table 2). On the other hand, there were no apparent differences in the relative changes in PT-INR between Plasma B and D, which had comparable PT-INR levels at baseline. For Reagent 2, there were no obvious differences between the plasma groups with a high baseline PT-INR (Plasma B, C, and D) and that with a normal baseline PT-INR (Plasma A): all groups responded to the similar extent.

## Discussion

We found a strong and significant linear association between daptomycin concentrations and the PT-INR prolongation in all the plasma groups and reagents. When the daptomycin concentration was higher than 100 μg/mL, PT-INR was prolonged by approximately 10–20%, particularly in those with elevated baseline PT-INR. These results are consistent with the findings in our previous in vivo study [7]. In our previous study, the linear association was observed only in Reagents 2 and 3. However, this result might be due to other clinical factors that can affect the vital coagulation system. Moreover, plasma samples with a high concentration (e.g., > 100 μg/mL) were limited in our previous study. Not all reagents used in this study have been tested in previous in vitro studies. In the present study, we confirmed the daptomycin concentration-dependent prolongation of PT-INR under controlled conditions.

In our previous study, peak daptomycin concentrations ranged from 30 to 90 μg/mL after the administration of 6 mg/kg of daptomycin [7]. Another study showed that daptomycin doses of more than 6 mg/kg led to higher peak daptomycin concentrations of more than 100 μg/mL [8]. Currently, the high doses of daptomycin are sometimes recommended for the treatment of serious infections. For example, 10 mg/kg of daptomycin is recommended for the treatment of persistent MRSA bacteremia [2]. We must take into consideration that severe infections caused by Gram-positive pathogens frequently occur in warfarin users because some of them have intra-cardiac devices, such as ventricular assist devices or prosthetic heart valves. Patients with intra-cardiac devices have higher PT-INR levels (up to 3.5) [9, 10]. If a serious infection caused by Gram-positive bacteria occurs in such patients, high doses of daptomycin will be administered, leading to a peak daptomycin concentration of more than 100 μg/mL. Consequently, PT-INR may be artificially prolonged by more than 10%, consequentially leading to an inappropriate reduction of the warfarin dose. We should ensure that PT is checked at the trough time of daptomycin concentrations.

For Reagents 1 and 3, the relative increase in PT-INR was higher in the plasma groups with a high baseline PT-INR (Plasma B, C, and D) than in the plasma group with a normal baseline PT-INR (Plasma A). The etiology of the coagulation abnormality also did not affect the relative change in PT-INR in all three reagents. These results can be explained by the fact that daptomycin interferes with phospholipids contained in the reagents rather than the coagulation factors in the plasma, which are decreased in both warfarin users and patients with liver cirrhosis. Although Webster et al. reported that prolonged baseline PT itself did not affect the relative change in PT, they lacked data on the relative change in PT in anticoagulated plasma samples with more than 100 μg/mL of daptomycin [3]. Our findings suggest that with high baseline PT-INR, it becomes more likely to observe clinically important relative change of >10% in PT-INR in at least some reagents.

Previous studies have shown that two factors in PT reagents may be responsible for the false PT prolongation: the thromboplastin source and the phospholipid type of reagents [3, 4]. It is conceivable that daptomycin that is inserted into the phospholipid component of PT reagents can interfere with tissue factor-induced coagulation, resulting in false PT prolongation [3]. Reagents made from recombinant human or rabbit tissue factors were highly susceptible to daptomycin when phosphatidylglycerol is added [4]. In this study, Reagents 1 and 3 were made from recombinant human tissue factors, although Reagent 2 was made from the rabbit brain. Therefore, the differences in the thromboplastin source might affect the susceptibility to daptomycin.

Our study had several limitations. First, we did not conduct statistical comparisons of the relative changes in PT-INR between the plasma groups due to the small sample sizes. In addition, biochemical studies are required to assess the mechanism by which daptomycin-induced artificial prolongation was larger in the plasma with elevated baseline PT-INR levels than in the normal plasma, regardless of the cause.

## Conclusions

In summary, we found that daptomycin induced the dose-dependent artificial prolongation of PT-INR in individuals with or without coagulation abnormalities. For some reagents, the extent of the relative change in PT-INR was higher in the plasma groups with a higher baseline PT-INR due to either warfarin or liver cirrhosis than in the plasma group with a lower PT-INR. The etiology of coagulation abnormalities did not apparently affect the relative change. The evaluation of PT-INR at the trough time of daptomycin must be considered, particularly in patients with a higher baseline PT-INR.

## Abbreviations

FDA: Food and drug administration
INR: International normalized ratio
LC: Liver cirrhosis
MRSA: Methicillin-resistant *Staphylococcus aureus*
PT: Prothrombin time
SD: Standard deviations
SE: Standard error
